# Pituitary Apoplexy (PA): Delayed Diagnosis of a Rare Clinical Syndrome in a Patient With a Known Pituitary Adenoma

**DOI:** 10.7759/cureus.31536

**Published:** 2022-11-15

**Authors:** Yusuf Mehkri, Emma Leone, Ramy Sharaf, Jairo Hernandez, Lorena Figueredo Rivas, Ibrahim S Tuna, Hans Shuhaiber

**Affiliations:** 1 College of Medicine, University of Florida College of Medicine, Gainesville, USA; 2 Neurology, University of Florida College of Medicine, Gainesville, USA; 3 Radiology, University of Florida College of Medicine, Gainesville, USA

**Keywords:** endocrinology, neurology case report, macroadenoma, cranial nerve deficit, pituitary apoplexy

## Abstract

Pituitary apoplexy (PA) is a rare clinical syndrome in which the pituitary gland undergoes infarction or hemorrhage, predominantly in the setting of an underlying tumor. We report on apoplexy of an expanding pituitary macroadenoma that was compressing the optic chiasm in a patient with progressively worsening neurologic deficits. Due to the patient's rapidly declining clinical status and family’s goals of care, no neurosurgical intervention took place, and the patient expired a few days following discharge to hospice. This case highlights the importance of early suspicion for apoplexy in a patient with a history of pituitary adenoma and signs of neurologic deficit.

## Introduction

Pituitary apoplexy (PA) is an acute life-threatening clinical emergency of the endocrine system, characterized by sudden hemorrhage or infarction of the pituitary gland [1]. PA is typically associated with the presence, and spontaneous rupture, of a pituitary adenoma, which can result in deficits to neurological and endocrine function [1]. Several different PA grading scales exist to characterize the degree of deficit resulting from the occurrence of PA [1]. Although PA occurs predominantly in patients with a pituitary adenoma, the incidence of PA within this patient population remains difficult to approximate, with a high variation in estimates ranging from 1% to 26% [1-4].

The most common early clinical manifestation of PA includes acute onset of severe headache with visual impairment, hemiparesis, nausea/vomiting, and/or ocular palsy [1, 3, 5]. More specifically, it is noteworthy to mention that PA is commonly associated with ocular palsy of the third cranial nerve (CN III) [5]. Hormonal and electrolyte disturbances following PA are not uncommon and must also be addressed rapidly [1]. In patients with a pituitary adenoma, additional risk factors for PA include hypertension, pregnancy, anticoagulation therapy, major surgery, and head trauma [2, 4-5]. It is noteworthy to mention that PA can still occur without the presence of any predisposing factors [3]. Rapid diagnosis and treatment of PA are critical; however, rapid diagnosis may be difficult given that the presence of a pituitary adenoma is unsuspected prior to the acute PA event in over 80% of patients [2, 5].

## Case presentation

This is a case of an 88-year-old male with a past medical history of pituitary adenoma, Alzheimer’s dementia, diabetes mellitus, stroke, myocardial infarctions, hypothyroidism, hypertension, hyperlipidemia, and chronic obstructive pulmonary disease who presented to our emergency department (ED) after falling backward attempting to ambulate from his bathroom.

The patient was diagnosed with a pituitary adenoma incidentally four years prior to evaluation for two episodes of syncope. Confirmed by both MRI and non-contrast head CT, the adenoma measured 1.7 cm x 1.2 cm x 1.3 cm in the right sellar region crossing midline. Additional findings at that time included stable encephalomalacia in the left temporal lobe with ex vacuo dilation of the left temporal horn and changes consist of advancing chronologic age such as mild ventriculomegaly, mild sulcal and cisternal prominence, senescent calcifications of the basal ganglia, and degenerative arthrosis of the atlantoaxial joint. At that time no acute intracranial edema, hemorrhage, herniation, or hydrocephalus was noted. Orbits, paranasal sinuses, and mastoid air cells were unremarkable. There were no visual field deficits revealed by cranial nerve examination and laboratory testing revealed as follows: decreased thyroid stimulating hormone (TSH) (<0.030), insulin-like growth factor (IGF-1) within normal limits (46), prolactin within normal limits (8.5), follicle stimulating hormone (FSH) within normal limits (5.4), luteinizing hormone (LH) within normal limits (4.5), and adrenocorticotropic hormone (ACTH) within normal limits (8). At the time of pituitary adenoma diagnosis, the patient refused intervention by neurosurgery. The adenoma remained grossly unchanged for the subsequent four years.

Upon presentation to our ED post-fall, the patient denied any memory of falling and could not elaborate on the details of the event, but on further questioning thought he had blacked out. The patient’s wife supplemented the story, explaining that the patient passed out that morning and hit his head in the process. The patient had not been acting himself over the prior few days, was less active than usual, and had been more forgetful. A conversation with the patient’s daughter corroborated this account; the patient had a new/worsening from baseline with slurred speech for the 6 days prior to ED presentation and a severe headache the day prior. Additionally, the patient had reported chest pain the day prior while sitting in his chair. 

At the time of presentation, the patient was in no acute distress but complained of minor head, shoulder, and back pain. The patient was also having difficulty speaking and was confused and anxious. His blood pressure was elevated (207/78) and all other vital signs were within normal range. On physical exam, there was tenderness over the thoracic spine and left shoulder, but no neurologic deficits were noted. At this time, the differential diagnosis included cardiac dysrhythmia, syncope due to orthostatic hypotension, seizure, hypoglycemia, and stroke. A complete workup was conducted.

**Figure 1:**
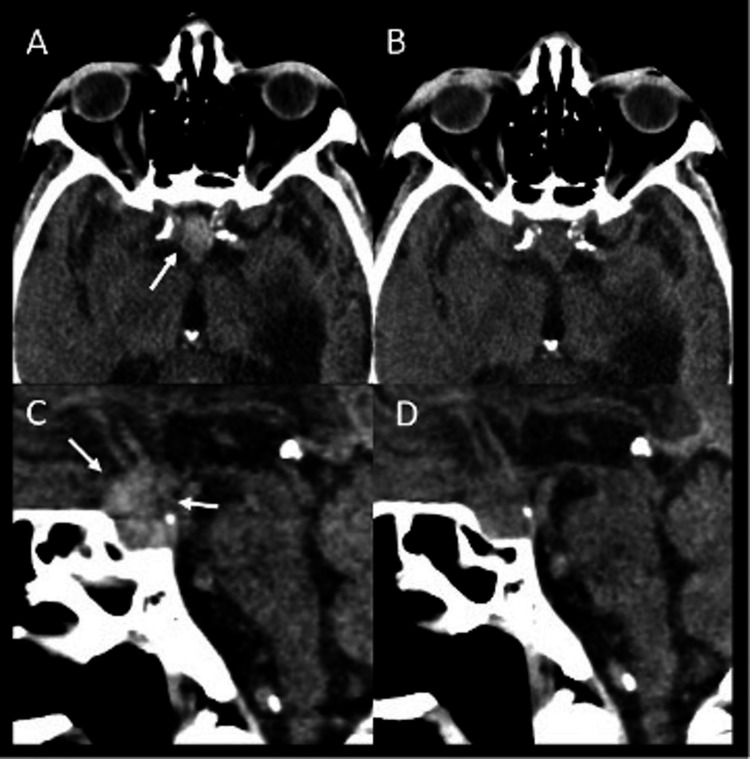
Unenhanced CT images in axial (A,B) and sagittal (C,D) plane demonstrates pituitary adenoma with interval development of hyperdensity within the lesion (arrows in A,C) compared to prior CT 2 weeks prior (B,D) compatible with interval pituitary hemorrhage.

The patient was administered 10 mg IV hydralazine with improvement (blood pressure, BP 192/71). Labs were notable for a mild acute kidney injury in the absence of leukocytosis, anemia, and urinary tract infection. Troponins were low and not rising. CT head (Figure 1) and C spine showed no acute changes, stable encephalomalacia in the left temporal lobe with resultant asymmetric ex vacuo dilatation of the left lateral ventricle, stable sellar mass extending toward the right cavernous sinus compatible with macroadenoma, intracranial atherosclerosis, and severe degenerative changes. Acute traumatic injury to the brain and cervical spine were ruled out. ECG showed a first-degree atrioventricular (AV) block.

X-ray of the lumbar and thoracic spine returned normal, except for degenerative joint disease. Chest and shoulder X-rays showed no acute cardiopulmonary process.

At this time, the patient was admitted for syncope, hypertensive emergency, and acute kidney injury. His syncopal episode was thought attributable to first-degree AV block. A transthoracic echocardiogram (TTE) was ordered as well as daily monitoring of the basic metabolic panel (BMP) to assess for electrolyte derangements. The patient was started on his home medicine routine and continued as needed hydralazine was given possible AV block. The patient’s blood pressure remained elevated at 189/68 throughout the course of hospital day 1.

Overnight from hospital day 1 to hospital day 2 the patient was hypertensive (BP 173/81), tachycardic (pulse 111), febrile (101.6 F), and mildly tachypneic. These values raised concern for sepsis of unknown source, along with possible hypovolemia. Preliminary infectious work-up at this time noted developing leukocytosis, therefore, empiric broad antibiotic coverage was initiated with vancomycin and cefepime. IV fluid resuscitation took place as well. Chest X-ray demonstrated increased bibasilar atelectasis and otherwise no new abnormalities.

On hospital day 3, the patient was afebrile and stable (BP 132/84, pulse 85) and antibiotics were discontinued. Physical exam remained within normal limits.

On hospital day 4, the patient became hypotensive (BP 78/44) and febrile (103.1 F) and on the physical exam, he had a right eye “sluggishness.” Differential diagnosis at this time expanded to concern for sepsis with no clear source identified, hypercapnic respiratory failure, cerebral edema/bleed, and medication/substance-induced hypotension. Arterial blood gases, blood cultures, urinalysis, ammonia levels, head CT, electroencephalogram (EEG), and lower and upper extremity venous ultrasound were ordered. The patient was administered Narcan, 1000 cc IV fluids, and restarted on cefepime and vancomycin with the return to normotensive status.

Labs returned concerning about hyponatremia (135), hypokalemia (3.2), and hyperglycemia (145). The white blood cell (WBC) count remained elevated (10.5) and platelet count dropped to 134. Cultures returned negative. EEG was reported negative for epileptogenic activity or seizures but was abnormal due to diffuse moderate slowing with frequent generalized rhythmic delta waves and moderate focal disturbance of cerebral activity congruent with a documented diagnosis of Alzheimer’s disease. Deep venous thrombosis was ruled out via negative venous ultrasounds.

Hospital day 5 was unremarkable, with lab work-up (TSH 0.491; C-reactive protein, CRP 139.86), cultures, and imaging unrevealing. The patient was normotensive and afebrile with a normal physical exam aside from his continued right eye sluggishness. 

On hospital day 6, the patient remained afebrile with continuing altered mental status, lethargy, and an episode of vomiting a coffee ground-like material. His right eye was sluggish but the rest of his physical exam remained within normal limits. Infectious disease (ID) was consulted for evaluation of the patient’s fever of unknown origin. It was noted that the patient had numerous mosquito bites and possible tick exposure as well as intermittent repeated myoclonic movements of his shoulders. Concern for viral encephalitis was addressed with a work-up. Broad-spectrum antibiotics were discontinued and the patient was started on acyclovir and doxycycline empirically.

On hospital day 7, the patient was mildly hypertensive (140/59), remained afebrile, and was refusing nutrition. His right eye continued to be sluggish. ID was consulted again. The patient was continuing to experience myoclonic movements of the shoulders and was arousable, but would immediately become unresponsive again Seizure activity and a post-ictal state were of concern. Upon discussion with the patient’s wife, it was disclosed that the patient had developed multiple brown macules on his back and shoulder at the time that the patient had become ill. A dermatology consult for the macular rash and a neurology consult for EEG for possible seizure activity were ordered.

**Figure 2:**
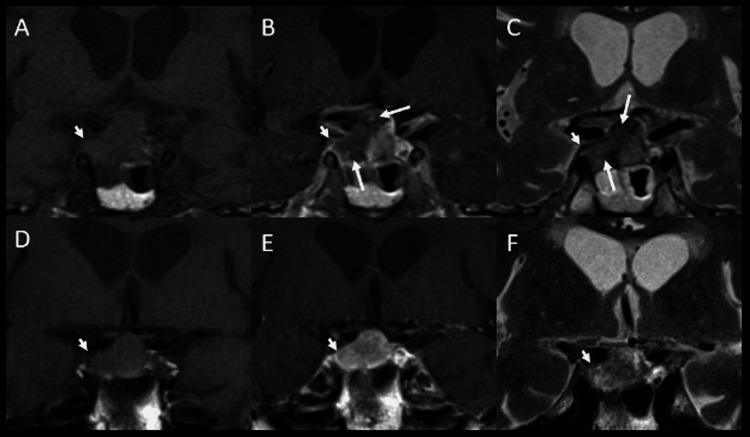
MRI sella with pre-contrast T1 (A,D), postcontrast T1 (B,E), and T2 (C,F) weighted imaging with comparison MRI from four years earlier (D,E,F) demonstrate pituitary macroadenoma with suprasellar and right cavernous sinus extension (arrowheads). There is development of heterogenous enhancement with a large non-enhancing component corresponding to interval necrosis and hemorrhage within the adenoma compatible with pituitary apoplexy (arrows in B). Changes in T2 signal with hypointensity (arrows in C) is due to evolving blood products.

On hospital day 8, due to the patient’s continued waxing and waning mental status and jerking movements in bilateral shoulders, an MRI was obtained, with findings noted in Figure 2. 

Neurosurgery was consulted and on a neurologic exam, the following was noted:

The patient could open his left eye to voices, but not his right. He would squeeze his right eye shut when the examiner attempted to open it. Pupils were asymmetric and sluggishly reactive (4 mm and 2 mm in oculus dexter, OD and oculus sinister, OS respectively). The patient was oriented to self and location, but not year. He could mimic speech, but was confused, mumbled incomprehensibly, and was dysarthric. He could follow simple commands in all extremities and could move all four spontaneously and symmetrically.

Neurology was also consulted and on a neurologic exam, the following was noted:

Glasgow Coma Scale (GCS) was valued at 9: R eye opened to pain (2), the verbal response was incomprehensible (2), and motor response localized to pain (5). The patient was confused with pupils 2 mm, equal, and reactive to light. Vestibulo-ocular reflex and corneal reflex were both present and facial movement was symmetric. The patient withdrew to pain in the upper and lower extremities with 2+ upper extremity reflexes bilaterally. The patient was unable to open both eyes, concerning third nerve palsy which could be caused by PA and hemorrhage.

The WBC remained elevated (10.3) and hemoglobin (HGB) (12.4) and hematocrit (HCt) (35.5) were low. Pituitary labs showed decreased IGF-1 (19), TSH (0.136), LH (0.9), and ACTH (<1.5); prolactin (7.6) and FSH (2.5) were within normal limits. Plasma glucose was elevated (185).

**Figure 3:**
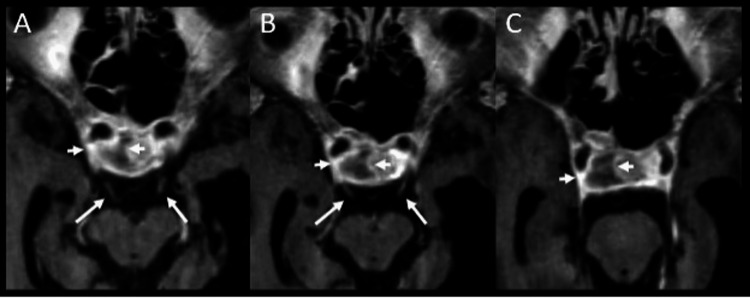
Sequential axial 3DT1 postcontrast imaging at the level of midbrain demonstrates heterogeneously enhancing pituitary adenoma with non-enhancing necrotic/hemorrhagic component extending to the right cavernous sinus (arrowheads). The cisternal segment of the CN3 seen in the interpeduncular and suprasellar cistern (arrowheads A,B) coursing to the right cavernous sinus where there is extension of the pituitary apoplexy.

Brain MRI with magnetic resonance angiography (MRA) was ordered to rule out any other etiology than PA and hemorrhage which could explain the patient’s third nerve palsy. MRI returned positive for hemorrhage and expansion within the pituitary tumor with compression of the optic chiasm/tracts (Figure 3). 

The patient’s family was informed of the MRI findings and was told that this would be an indication for surgery. The family reiterated the patient’s unwillingness to undergo neurosurgery, stating that the patient would “never want surgery on his head” and had made this statement many times before. The family understood that, without surgery, the patient could become blind, have other neurologic or hormonal issues, and possibly expire. The family voiced understanding of these potential risks of not undergoing surgery and maintained that the patient would still never want surgery, even if vision-saving or life-saving. The patient’s code status was DNR and he was subsequently not treated with surgery and placed on seizure prophylaxis with levetiracetam.

On hospital day 9, the patient had minimal alertness, did not open his eyes to voice, did not localize towards the examiner, and did not follow commands. Pupils were 3 mm oculus uterque (OU) and reactive, blinking tolerance time (BTT) was absent bilaterally, cranial nerve III palsy on the right with complete ptosis and extraocular muscle (EOM) paresis were noted, and there was no spontaneous nystagmus. Corneal reflex was present, as was cough reflex. Spontaneous movements were absent, no abnormal/myoclonic movements were noted, and muscle tone and reflexes were normal throughout. Based on anticipation of transferring the patient to palliative care, conservative management was begun, including levetiracetam and delirium precautions. The patient was discharged to hospice in stable condition and expired five days later.

## Discussion

The presence of a pituitary adenoma is typically the single greatest underlying risk factor contributing to the occurrence of PA [4, 5]. While there remains uncertainty regarding the exact pathophysiology of PA, investigation of the underlying pathophysiology of pituitary adenomas may lend to a greater understanding of the cellular and biochemical processes involved in PA [4]. There are the two main binary classifications of pituitary adenomas - namely, functional vs. non-functional adenomas, and microadenomas vs. macroadenomas, based on hormonal secretion activity and tumor size respectively. Although there are inconsistencies in the literature surrounding the differential incidence of PA associated with the various types of pituitary adenomas, PA is more common in patients with a pituitary macroadenoma [2-4, 6]. Patients with a pituitary macroadenoma also more commonly exhibit long term pituitary hormone deficiencies following PA [7].

Pituitary adenomas are typically benign; however, their high metabolic requirements coupled with limited vascularization and poor angiogenesis increases their susceptibility to hemorrhage and infarction [4, 8]. Pituitary adenomas are thus sensitive to changes in perfusion and several of the precipitating risk factors for PA are those which may affect hemodynamic stability or increase intratumoral or intrasellar pressure [1, 4, 8-10]. Although asymptomatic hemorrhage of a pituitary adenoma is not uncommon, hemorrhage or infarction of a pituitary adenoma typically occurs acutely, resulting in sudden onset of severe headache [3]. Expansion along the superior border of a pituitary adenoma is possible following hemorrhage or infarction of the tumor, which can compromise the optic nerve, optic chiasm, or optic tract and may contribute to neurological, oculomotor, and/or visual field defects [9, 11]. Given the anatomical location of the pituitary gland, expansion of the tumor can result in third nerve palsy - either by way of decreasing vasculature to the nerve or by direct compression [11].

Delayed diagnosis of PA is common given that the underlying pituitary adenoma is commonly undiagnosed in patients prior to the acute PA event [1, 4]. As such, PA should be considered in patients presenting with acute onset of severe headache. When PA is suspected, anterior pituitary hormone levels must be examined as deficiencies in these hormones have been evidenced to occur in nearly 80% of PA cases [4, 12, 13]. For patients in whom PA is suspected, MRI or CT imaging is required to visualize whether hemorrhage or infarction of a pituitary adenoma has occurred so that a definitive diagnosis can be made [4, 12]. MRI is typically preferred over CT imaging in diagnosing PA given its higher sensitivity; however, CT angiography or Digital Subtraction Angiography can be used to exclude occurrence of an intracranial aneurysm which may present similarly to PA [4, 12, 14]. Although treatment and management of PA has remained highly controversial, a conservative management approach has been indicated to produce favorable outcomes in PA patients presenting without visual field deficits [4, 5]. The conservative management approach of PA includes a combination of hydrocortisone administration, and supportive measures to restore fluid, electrolytic, and hemodynamic stability [5]. If the conservative management approach is indicated, close monitoring of the patients neurological, visual, and endocrine functions is required [5]. In patients experiencing ocular palsy, visual field deficits, or deterioration of consciousness secondary to PA, surgical resection of the adenoma may be required to decompress and thus restore proper ophthalmic and neurological function [4, 9, 11, 14]. For this patient population, early surgical intervention has been evidenced to contribute to optimal restoration of visual acuity [5, 11].

Our patient presented with complaint of severe headache, recent head trauma, confusion and difficulty speaking. With a history of pituitary adenoma and stroke, head imaging and pituitary hormone level work-up should have been priority. On day 4, sluggish eyes were recorded, and concern for neurologic pathology again should have been at the top of the differential. Instead, there was a delay in diagnosis and the patient’s status rapidly declined by day 8 and intervention was declined in place of comfort care. In one institutional review of 33 patients presenting with pituitary apoplexy, over 80% presented with headache, 50% with hormonal deficiencies, and about 40% with visual disturbances [15]. All patients had characteristic findings on MRI. In a separate retrospective review of 30 patients with acute pituitary apoplexy, recent fluctuations in blood pressure and anti-coagulation status were associated with apoplexy [16]. These were also significant factors in our patient’s case. Ultimately, this outcome was likely avoidable and highlights the importance of rapid and appropriate clinical decision making when working up a patient with these presenting symptoms and a history of pituitary adenoma.

## Conclusions

Delayed diagnosis of PA is common in the case of undiagnosed pituitary adenoma but can be avoided in a patient with a known diagnosis given key presenting symptoms. In our patient, the concern for recent head trauma, severe headache, fluctuations in blood pressure, visual disruptions and altered mental status should have raised concern for neurologic pathology and immediate MRI. Although the underlying cause was eventually isolated, the differential diagnosis included cardiac dysrhythmia, syncope due to orthostatic hypotension, seizure, hypoglycemia, and stroke, with significant delay in diagnosis of the apoplexy and resulting unfavorable outcome.
